# A Novel Combination of Serum Markers in a Multivariate Model to Help Triage Patients Into “Low-” and “High-Risk” Categories for Prostate Cancer

**DOI:** 10.3389/fonc.2022.837127

**Published:** 2022-05-19

**Authors:** Christopher J. McNally, Joanne Watt, Mary Jo Kurth, John V. Lamont, Tara Moore, Peter Fitzgerald, Hardev Pandha, Declan J. McKenna, Mark W. Ruddock

**Affiliations:** ^1^Genomic Medicine Research Group, Ulster University, Coleraine, United Kingdom; ^2^Clinical Studies Group, Randox Laboratories Ltd., Crumlin, United Kingdom; ^3^Royal Surrey County Hospital NHS Foundation Trust, Research Development and Innovations Department, The Royal Surrey County Hospital, Guildford, United Kingdom; ^4^School of Biosciences and Medicine, Faculty of Health and Medical Sciences, University of Surrey, Guildford, United Kingdom

**Keywords:** algorithm, EGF, fPSA, IL-8, marker, MCP-1, prostate cancer, tPSA

## Abstract

**Background:**

Almost 50,000 men in the United Kingdom (UK) are diagnosed each year with prostate cancer (PCa). Secondary referrals for investigations rely on serum prostate-specific antigen (PSA) levels and digital rectal examination. However, both tests lack sensitivity and specificity, resulting in unnecessary referrals to secondary care for costly and invasive biopsies.

**Materials and Methods:**

Serum samples and clinical information were collected from *N* = 125 age-matched patients (*n* = 61 non-PCa and *n* = 64 PCa) and analyzed using Biochip Array Technology on high-sensitivity cytokine array I (IL-2, IL-4, IL-6, IL-8, IL-10, IL-1α, IL-1β, TNFα, MCP-1, INFγ, EGF, and VEGF), cerebral array II (CRP, D-dimer, neuron-specific enolase, and sTNFR1), and tumor PSA oncology array (fPSA, tPSA, and CEA).

**Results:**

The data showed that 11/19 (68.8%) markers were significantly different between the non-PCa and the PCa patients. A combination of EGF, log_10_ IL-8, log_10_ MCP-1, and log_10_ tPSA significantly improved the predictive potential of tPSA alone to identify patients with PCa (DeLong, *p* < 0.001). This marker combination had an increased area under the receiver operator characteristic (0.860 *vs*. 0.700), sensitivity (78.7 *vs*. 68.9%), specificity (76.5 *vs*. 67.2%), PPV (76.2 *vs*. 66.7%), and NPV (79.0 *vs*. 69.4%) compared with tPSA.

**Conclusions:**

The novel combination of serum markers identified in this study could be employed to help triage patients into “low-” and “high-risk” categories, allowing general practitioners to improve the management of patients in primary care settings and potentially reducing the number of referrals for unnecessary, invasive, and costly treatments.

## Introduction

Prostate cancer (PCa) is very common, with almost 50,000 men diagnosed each year in the UK (1) and 240,000 in the US (2). Annually, PCa kills almost 35,000 men in the US (2). Tumors of the prostate are likely to be localized, clinically unapparent, and with International Society of Urological Pathology (ISUP) grade grouping 1 (3, 4). Slow-growing, non-significant PCa may not cause serious harm (5) and often does not require any intervention. However, clinically significant prostate cancers require urgent treatment, as they have the potential to metastasize and cause a serious disease.

Patients with PCa are usually asymptomatic, and the presenting symptoms are not specific and are often observed in men with benign prostate enlargement (BPE), one of the most frequently reported age-related diseases in men over 60 years. The symptoms include painful or burning sensation during urination, frequent urination (particularly at night—nocturia), difficulty stopping and starting urination, sudden erectile dysfunction, blood in the urine (hematuria) or semen, bone pain, and weight loss.

The risk factors for PCa include patient age (>50 years), ethnicity (African-American ethnicity and other minority ethnicities have a greater risk of progression and are more likely to develop aggressive cancer than Caucasian men), obesity (patients who are obese have a higher risk of PCa), and family history (blood relative, *e*.*g*., parent) (6). The complications of PCa and subsequent treatment include metastatic spread of the disease, urinary incontinence, and erectile dysfunction (7).

The gold standard for diagnosing PCa is histological assessment of prostate tissue obtained by transrectal ultrasound-guided systematic (TRUS) core needle biopsy. The most common scale used to evaluate the grade of PCa is the Gleason score (8). The higher the Gleason score, the more likely that the cancer will grow and spread quickly (9).

Screening patients for PCa remains controversial and is not recommended due to the potential for overtreatment (10). Data presented by the Surveillance, Epidemiological, and End Results registry have estimated that screening for PCa, using prostate-specific antigen (PSA) alone, resulted in an increase of 28% of patients being over-diagnosed in the US (11). Furthermore, the European Randomised Study of Screening for Prostate Cancer trial also estimated that, when PSA is used alone as a screening tool for PCa, almost 50% of patients were over-diagnosed (12).

Although advances in PCa management have been made, an elevated PSA and/or abnormal digital rectal examination (DRE; nodular, indurated, and/or asymmetry) would still normally warrant a referral for investigation (13). An abnormal DRE is the second most common finding that initiates further investigation for malignancy (14–16). As a result, many patients with elevated PSA/abnormal DRE are referred to secondary care for invasive and costly procedures (17). These are often unnecessary as almost 75% of patients who are referred for further investigation have a negative biopsy (18). In addition, some 2.5 to 3% of patients are admitted to a hospital within a week of their TRUS procedure with a serious infection (urinary tract infections and/or bacterial prostatitis). This could be avoided with better decision-making in primary care but requires more biological information on the patient’s disease to be available to their GP.

Currently, no biomarker or biomarker combinations that have the sensitivity and specificity to replace PSA have been identified (19). Therefore, improved approaches are required to differentiate between men who have a prostate disease that require treatment or surveillance and those who do not. The symptoms and PSA results are not an accurate indicator of disease. Indeed no level of PSA is truly diagnostic (20)—for example, a patient could have a PSA >10 ng/ml and not have any cancer, whereas another patient with a PSA <1 ng/ml could have aggressive cancer. Therefore, there is an urgent need for new tests which can at least stratify patients and, if possible, be diagnostic. However, it is very unlikely, given the heterogeneous nature of PCa, that a single biomarker will prove to be diagnostic.

The effective management of PCa requires an accurate diagnosis. However, the challenge for the clinician is to differentiate benign conditions (BPE) from PCa, which presents with similar symptoms. The PSA test exhibits a negative benefit-to-harm ratio based on population estimates (12). Therefore, biomarkers that would contribute to the sensitivity and specificity of PSA could offer the clinician additional information so that a more informed management decision could be made on whether to refer a patient to secondary care for further investigations or to manage the patient in primary care.

The aim of the study was to investigate the levels of serum markers in patients who present to primary care with PCa-like symptoms so as to identify markers that could be used to improve the triage of patients into low- and high-risk categories, thereby enhancing patient management.

## Materials and Methods

### Patient Cohort and Sample Collection

One hundred twenty-five patients were included in the study. The patient cohort consisted of two independent patient sample sets.

The first set of patients (*N* = 33; *n* = 10 non-PCa and *n* = 23 PCa) were recruited by Royal Surrey County Hospital (NHS Foundation Trust) between 2015 and 2018 (Diagnosis of Clinically Significant Prostate Cancer; Royal Surrey County Hospital, Research Development and Innovations Department, The Royal Surrey County Hospital, Leggett Building, Daphne Jackson Road, Guildford, Surrey GU2 7WG, 15/LO/0218). The inclusion criteria included (i) men >18 years referred by their GP to investigate the cause of (ii) an abnormal PSA test. The exclusion criteria included (i) an active urine infection, confirmed by urine dipstick testing or midstream urine microscopy, (ii) men with a PSA <4 and >20 ng/ml, (iii) men already diagnosed with PCa, (iv) men with a prior or concurrent malignancy (apart from basal cell carcinoma of the skin), and (v) men who cannot give informed consent (**Supplementary 1**). Blood (24 ml) and urine (20–30 ml) were collected after a prostatic examination, along with a detailed clinical history. The study complied with the Declaration of Helsinki, and written informed consent was obtained from all participants.

The second patient cohort (*N* = 92; *n* = 54 non-PCa and *n* = 38 PCa) was obtained from Discovery Life Sciences (DLS; CA, USA). The patient samples were de-identified and publicly available and were thus exempt from the requirement of the Institutional Review Board (IRB) approval (exempt category 4, IRB/EC). However, the DLS samples were procured pursuant to informed consent provided by the individual under approved protocols 45 CFR 46.116. Serum (1 ml) with clinical history was obtained for each DLS patient. The samples were selected from treatment-naive patients based on ICD-10 codes for prostate-related conditions.

### Pathological Examination of Prostate Biopsies

Prostate cancer was confirmed by a histological examination of prostate biopsies from both sample sets. The Gleason scores assigned by the pathologists are described in **Table 1**. The non-PCa group included patients with confirmed benign prostatic hyperplasia (BPH; *n* = 30/61, 49.2%). All patients were treatment-naïve at the time of prostate biopsy.

**Table 1:** Clinical and pathological characteristics of the patients. Data shown as mean ± SD or *n*/total (%), Wilcoxon rank-sum test; *p <* 0.05 was considered significant.

Clinical characteristics	Non-PCa (*n* = 64)	PCa (*n* = 61)	*p*-value
Age (years)	62.7 ± 10.4	64.4 ± 8.3	0.439
BPH	30/64 (46.9%)		
**Gleason score**			
6		11/60 (18.3%)	
7		31/60 (51.7%)	
8		12/60 (20%)	
9		6/60 (10%)	
tPSA (ng/ml)	4.2 ± 3.7	20.8 ± 58.2	<0.001
fPSA (ng/ml)	0.8 ± 0.9	3.6 ± 9.5	0.005
CEA (ng/ml)	2.4 ± 3.0	4.4 ± 16.5	0.158

PCa, prostate cancer; BPH, benign prostatic hyperplasia; tPSA, total prostate-specific antigen; fPSA, free prostate-specific antigen; CEA, carcinoembryonic antigen.

Both patient cohorts were combined (*N* = 125) and separated into two groups, depending on the pathology reports: non-PCa (*n* = 64/125, 51.2%) and PCa (*n* = 61/125, 48.8%).

### Clinical Factors and Behaviors

Clinical factors were not available for all patients. However, where data was available, the most common presenting symptoms included the following: lower urinary tract symptoms (LUTS), urine retention, urgency, nocturia, lower back pain, and microscopic hematuria. For many of the patients, there was no previous history of benign disease prior to their PCa diagnosis.

Smoking history and details on alcohol consumption (units/week) were also available for a limited number of patients. Many PCa patients were former smokers. Where data was available, the number of cigarettes smoked per day ranged from 10 to 25. Pack-year data was not available. The alcohol consumption ranged from 1 to 48 units/week (where data was available).

Medications were also noted for a limited number of patients; where data was available, the most common drugs that the patients were prescribed with included sertraline, loratadine, omeprazole, aspirin, tamsulosin, simvastatin, losartan, atorvastatin, imvastatin, bendroflumethiazide, citalopram, sildenafil, fluoxetine, ranitidine, metformin, and bisoprolol.

### Biomarker Analysis

Patient blood and urine samples were stored in duplicate at -80°C prior to analysis by Randox Laboratory Clinical Services, Antrim, UK, by scientists blinded to the patients’ data. In total, 19 biomarkers were investigated by Biochip Array Technology (BAT) (Randox Laboratories Ltd., Crumlin, UK) (21) using the Evidence Investigator analyzer (Randox Laboratories Ltd., Crumlin, UK) and following the manufacturer’s instructions. The limits of detection (LOD) for the markers on the biochip arrays were as follows: EGF, 2.5 pg/ml; IFNγ, 2.1 pg/ml; IL-1α 0.9, pg/ml; IL-1β, 1.3 pg/ml; IL-2, 4.9 pg/ml; IL-4, 3.5 pg/ml; IL-6, 0.4 pg/ml; IL-8, 2.3 pg/ml; IL-10, 1.1 pg/ml; MCP-1, 25.5 pg/ml; TNFα, 3.7 pg/ml; VEGF, 10.8 pg/ml; CRP, 0.67 mg/L; D-dimer, 2.1 ng/ml; neuron-specific enolase (NSE), 0.26 ng/ml; sTNFR1, 0.24 ng/ml; CEA, 0.29 ng/ml; fPSA, 0.02 ng/ml; and tPSA, 0.045 ng/ml. The biomarkers below the LOD were recorded as 90% of the LOD (22).

### Statistical Analyses

Statistical analyses were undertaken using R version 4.0.5 (23). Wilcoxon rank-sum test was used to identify differentially expressed markers. Markers with *p <*0.05 were considered significant. The ability of the markers to predict PCa was further investigated using logistic LASSO regression following a cross-validation testing of several models. For marker and marker combinations, areas under the receiver operator characteristic (AUROC) (and 95% CI), sensitivity (and 95% CI), specificity (and 95% CI), positive predictive value (PPV), and negative predictive value (NPV) were calculated to identify models that differentiated between the two diagnostic groups (non PCa *vs*. PCa). DeLong test was used to compare AUROCs for the model and tPSA; *p <*0.05 was considered significant.

## Results

The clinical and pathological characteristics of the patients involved in the study are described in **Table 1**. Both tPSA and fPSA were significantly elevated in the PCa group. However, CEA was not significantly different.

### Biochip Array Technology

From the marker results obtained using the biochip arrays, 11/16 (68.8%) markers were significantly different between the non-PCa and the PCa patient groups (**Table 2**). Of these, 7/16 (43.8%) markers were elevated in the PCa patients *vs*. non-PCa, 4/16 (25%) were lower in the PCa *vs*. non-PCa, and 5/16 (31.2%) were not significantly different between either group.

**Table 2:** The analysis showed that 11/16 (68.8%) serum markers were significantly different between the non-PCa and the PCa patient groups.

Marker	non-PCa (*n* = 64)	PCa (*n* = 61)	*p*-value
IL-8 (pg/ml)	175.3 ± 261.5	28.4 ± 42.4	<0.001
IL-10 (pg/ml)	1.8 ± 2.0	3.2 ± 9.0	<0.001
MCP-1 (pg/ml)	189.9 ± 106.9	291.1 ± 148.0	<0.001
VEGF (pg/ml)	69.1 ± 68.5	145.5 ± 132.9	<0.001
IL-1β (pg/ml)	11.6 ± 44.1	1.9 ± 1.2	0.001
NSE (ng/ml)	15.3 ± 11.3	7.8 ± 5.3	0.001
EGF (pg/ml)	87.1 ± 54.7	129.5 ± 81.8	0.002
IL-6 (pg/ml)	37.8 ± 148.2	19.9 ± 42.1	0.004
sTNFRI (ng/ml)	1.2 ± 1.3	1.5 ± 1.1	0.009
CRP (μg/ml)	45.5 ± 41.0	73.8 ± 49.6	0.012
D-dimer (ng/ml)	173.6 ± 194.2	331.0 ± 382.9	0.014
IL-1α (pg/ml)	0.8 ± 0.1	0.8 ± 0.0	0.090
TNFα (pg/ml)	4.2 ± 3.1	3.9 ± 1.4	0.130
IL-2 (pg/ml)	4.7 ± 1.6	4.4 ± 0.1	0.327
IFNγ (pg/ml)	1.9 ± 0.2	1.9 ± 0.2	0.606
IL-4 (pg/ml)	3.2 ± 0.4	3.2 ± 0.4	0.608

Data shown as mean ± SD. Wilcoxon rank-sum test; p < 0.05 was considered significant.

PCa, prostate cancer; IL-8, interleukin-8; IL-10, interleukin-10; MCP-1, monocyte chemoattractant protein-1; VEGF, vascular endothelial growth factor; IL-1β, interleukin-1β; NSE, neuron-specific enolase; EGF, endothelial growth factor; IL-6, interleukin-6; sTNFR1, soluble tumor necrosis factor receptor-1; CRP, C-reactive protein; IL-1α, interleukin-1α; TNFα, tumor necrosis factor-α; IL-2, interleukin-2; IFNγ, interferon γ; IL-4, interleukin-4.

### Regression Analysis

**Figure 1:**
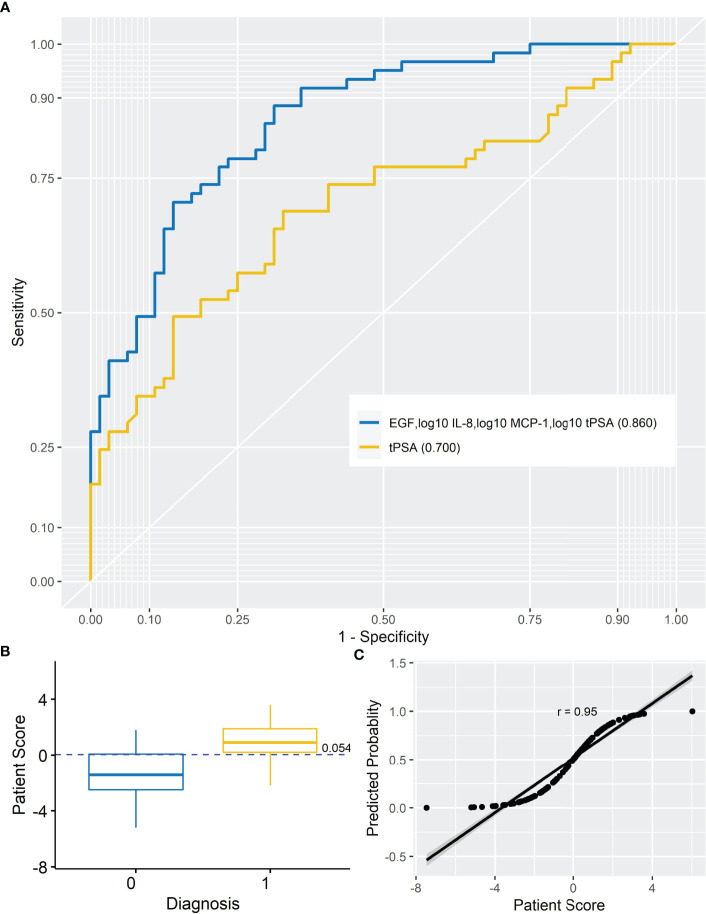
Prostate cancer model. **(A)** AUROC for analyte model (AUROC, 0.860) and tPSA (AUROC, 0.700). When the AUROC for the model (EGF, log_10_ IL-8, log_10_ MCP-1, and log_10_ tPSA) was compared with the AUROC for tPSA, the model significantly improved upon tPSA alone (DeLong, *p* < 0.001) at differentiating non-PCa from PCa patients. **(B)** Simple box plot of patient score by diagnosis [non-PCa (0) and PCa (1); mean ± SD] for the model at a cutoff of 0.054. **(C)** Simple scatter with fit line for predicted probability by patient score for the marker model (*r* = 0.95). AUROC, area under receiver operating characteristic; IL-8, interleukin-8; MCP-1, monocyte chemoattractant protein-1; EGF, endothelial growth factor; tPSA, total prostate-specific antigen.

Logistic LASSO regression identified a model for a combination of markers that demonstrated higher sensitivity and specificity *vs*. tPSA alone (**Table 3**). The four markers selected by LASSO regression to identify patients with PCa included EGF, IL-8, MCP-1, and tPSA (**Figure 1A**). As some of the data was not normally distributed, log_10_ transformation was applied to IL-8, MCP-1, and tPSA in the model.

**Table 3:** Individual analytes and model EGF, IL-8, MCP-1, and tPSA AUROC, sensitivity, specificity, PPV, and NPV for non-PCa *vs*. PCa.

Markers and marker combination	AUROC (95% CI)	Sensitivity (95% CI)	Specificity (95% CI)	PPV (%)	NPV (%)
EGF	0.658 (0.562–0.754)	0.656 (0.541–0.770)	0.609 (0.500–0.734)	61.5	65.0
IL-8	0.703 (0.612–0.794)	0.738 (0.623–0.836)	0.563 (0.438–0.688)	61.6	69.2
MCP-1	0.739 (0.651–0.826)	0.738 (0.623–0.836)	0.703 (0.594–0.813)	70.3	73.8
tPSA	0.700 (0.606–0.793)	0.689 (0.574–0.803)	0.672 (0.563–0.781)	66.7	69.4
EGF + log_10_ IL-8 + log_10_ MCP-1 + log_10_ tPSA	0.860 (0.796–0.923)	0.787 (0.688–0.885)	0.765 (0.656–0.875)	76.2	79.0

PCa, prostate cancer; IL-8, interleukin-8; MCP-1, monocyte chemoattractant protein-1; EGF, endothelial growth factor; tPSA, total prostate-specific antigen; AUROC, area under receiver operating characteristic curve; CI, confidence interval (95%); PPV, positive predictive value; NPV, negative predictive value.

When comparing the new model identified by LASSO to tPSA on its own, the number of false positives was reduced from 21/64 (32.8%) to 15/64 (23.4%), and the number of false negatives increased from 11/61 (18.0%) to 13/61 (21.3%) (**Table 4**).

**Table 4:** Confusion matrices comparing tPSA and the model EGF, IL-8, MCP-1, and tPSA.

		tPSA		Model	
		Predicted		Predicted	
		No PCa	PCa	No PCa	PCa
Actual	No PCa	43	19	49	13
	PCa	21	42	15	48

For each matrix, the figure in the top left represents the true number of negatives, the top right figure represents the number of false positives, the bottom left figure represents the number of false negatives, and the bottom right figure represents the number of true positives.

PCa, prostate cancer; IL-8, interleukin-8; MCP-1, monocyte chemoattractant protein-1; EGF, endothelial growth factor; tPSA, total prostate-specific antigen.

### Calculating the Patient Risk Score

The risk of PCa was based on the following marker combination: EGF, log_10_ IL-8, log_10_ MCP-1, and log_10_ tPSA. In this dataset, a cutoff of 0.054 (as shown in **Figure 1B**) was applied to achieve the highest sensitivity and specificity for identifying patients with PCa; PRS <0.054—patients are negative for PCa, whereas PRS ≥0.054—patients would be positive for PCa. It should be noted that the PRS would be used in combination with clinical risk factors when triaging patients. Thus, patients with a positive risk score and positive clinical risk factors (*e*.*g*., painful or burning sensation during urination, frequent urination, difficulty starting or stopping urination, sudden erectile dysfunction, and blood in urine or semen) could be prioritized for urgent referral for further investigations. Patients who were positive for clinical risk factors and negative for marker risk (PRS) could potentially be managed in primary care or referred for investigation as necessary. Importantly, this type of combined measurement approach is recommended for risk stratification methods by the National Institute for Health and Care Excellence (NICE 2019) guidelines for PCa.

To test the linearity of the model, predicted probability was plotted against patient score (**Figure 1C**). The high correlation between the predicted probability and patient score (*r* = 0.95) would suggest confidence in the model.

## Discussion

In this study, we investigated 19 serum markers involved in PCa (**Supplementary 2**). The results showed 11/16 (68.8%) cytokines that were significantly different between the non-PCa *vs*. PCa groups. Seven of these markers were elevated in the PCa group, whereas 4 markers were elevated in the non-PCa group. In the PCa group, 2/3 (66.6%) cancer markers (fPSA and tPSA) were also elevated.

The serum levels of IL-10, EGF, VEGF, MCP-1, sTNFR1, CRP, and D-dimer were significantly higher in the PCa patients. Of these serum markers, MCP-1 had the highest AUROC for detecting PCa (MCP-1 0.739 *vs*. tPSA 0.700). MCP-1 (CCL2) is a member of the chemokine family that acts as a paracrine and autocrine factor to promote PCa growth and invasion (24). MCP-1 is also a potent chemotactic factor regulating stromal–epithelial cells in PCa (25). Unsurprisingly, the angiogenic factors VEGF and EGF were also elevated in patients with PCa. However, in other studies, VEGF has been shown to have no significant prognostic or predictive value for expression for localized or advanced PCa (26). In contrast, EGF modulates PCa invasiveness by regulating the urokinase-type plasminogen activity (27). Inhibition of the EGF receptor may prevent tumor cell dissemination (28).

CRP is a general marker for inflammation, although it does not differentiate benign from malignant disease (29). However, IL-10, which was also elevated in our PCa patients, has anti-inflammatory and anti-angiogenic properties (30). Therefore, it was unsurprising that both markers were elevated in the PCa patients.

The thrombotic factor D-dimer has been detected in patients with PCa. However, the relationship between PCa and the coagulation disorder remains unknown (31). Nonetheless, high plasma levels of D-dimer are associated with an increased risk of PCa mortality (32). Similarly, sTNFR1 has been identified in men with PCa. Furthermore, sTNFR1 has been shown to be a potential biomarker for identifying PCa when compared with PSA alone (AUROC 0.97) (33). However, as this was a small study, the authors acknowledged that the results need to be assessed in a much larger patient cohort. In our study, sTNFR1 had an AUROC of 0.635 for PCa.

Prostate cancer is an inflammatory disease; however, we found that 4/11 (36.4%) inflammatory markers (IL-8, IL-1β, NSE, and IL-6 levels) were significantly lower in the PCa patients.

The circulating IL-8 serum levels have not been shown to be a significant predictor of diagnosis, aggressiveness, or prognosis for PCa (34). However, increased circulating IL-8 serum levels have been detected in patients with an underlying inflammatory disease (34). In our study, IL-8 was identified as a marker that could differentiate non-PCa from PCa, potentially by identifying patients with inflammatory disease, *i*.*e*., BPH. In addition, IL-1β is elevated in patients with chronic prostatitis, chronic pelvic pain syndrome, and BPH (35, 36). Furthermore, elevated IL-6 has also been reported in men with BPH, LUTS, and erectile disfunction (37). Therefore, it was not surprising that these three markers were elevated in the non-PCa patient group; almost 50% of non-PCa patients had a diagnosis of BPH.

Higher levels of NSE have been observed in non-PCa patients (38), albeit higher levels of NSE have also been observed in patients with metastatic disease (39). In our study, 42/64 (65.6%) PCa patients had a Gleason score ≤7; no information was available on metastatic disease, and there was no significant difference in the NSE levels by Gleason score (data not shown). However, elevated serum NSE has been suggested to correlate with prognosis in advanced PCa (38). The PCa patients in our study were treatment-naïve, and only 6/64 (9.4%) patients presented with a Gleason score ≥9.

In our study, the serum levels of IL-1α, TNFα, IL-2, IL-4, and IFNγ were not significantly different between the non-PCa and the PCa groups.

### Combination Model

The results demonstrated that no single marker significantly outperformed tPSA. However, a combination of EGF, log_10_ IL-8, log_10_ MCP-1, and log_10_ tPSA significantly improved the predictive potential of tPSA alone to identify patients with PCa. This marker combination had an increased AUROC (0.860 *vs*. 0.700), sensitivity (78.7 *vs*. 68.9%), specificity (76.5 *vs*. 67.2%), PPV (76.2 *vs*. 66.7%), and NPV (79.0 *vs*. 69.4%) compared with tPSA.

Using this marker combination in this patient dataset reduced the number of false positives from 21/64 (32.8%) to 15/64 (23.4%); however, the number of false negatives increased from 11/61 (18.0%) to 13/61 (21.3%) compared with tPSA. Thus, an additional 9.4% (6/64) of patients were correctly assigned as non-PCa using the marker combination. If the management of these patients was based solely on their tPSA results, *n* = 7 patients could have potentially undergone unnecessary and invasive investigations. An additional 3.3% (2/61) of patients were incorrectly assigned as PCa.

Evidence suggests that the use of multiple markers to differentiate non-clinically significant from clinically significant disease is an important strategy for reducing unnecessary referrals for further investigation (40). Integrating inflammatory serum biomarkers into a risk calculator may provide additional information for detecting and managing PCa risk (40). The predictive value of inflammatory markers for PCa diagnosis has been evaluated in primary care (41). Our data demonstrate the value of measuring multiple markers in this heterogenous pathophysiology in combination with tPSA. The main limitations of this feasibility study included the following: (1) the small number of participants in each patient cohort and (2) the limited patient information [demographics, behaviors, medications, socioeconomic data, and clinicopathological data (*e*.*g*., DRE)]. Nevertheless, these results warrant further investigation in a larger cohort, and this will help validate the model.

It is worth noting that other combination models are being investigated elsewhere, including the Stockholm-3 risk-based model (42), the 4kscore (43), the European Randomised Study of Screening for Prostate Cancer risk calculator (44), the Prostate Cancer Prevention Trial (45), and the Irish Prostate Cancer Risk Calculator (46). The work described in this study is therefore an important addition to the global research effort to identify combinations of biological and clinical measurements to inform evidence-based decision-making in PCa patients.

## Conclusion

This study demonstrated that a novel serum marker combination of EGF, log_10_ IL-8, log_10_ MCP-1, and log_10_ tPSA significantly improved the predictive potential of tPSA alone to identify patients with PCa. Application of this serum marker combination could provide clinicians with valuable information to help triage their patients into low- and high-risk categories. Improved risk category stratification of patients would enable better management of men who present at primary care with prostate-cancer-like symptoms. In turn, the utilization of this novel combination of markers could potentially reduce the number of patients that are referred to secondary care for unnecessary, costly, and invasive procedures. However, it should be noted that this is a preliminary study and the markers identified would need to be validated in a larger patient cohort.

## Data Availability Statement

The raw data supporting the conclusions of this article will be made available by the authors without undue reservation.

## Ethics Statement

The studies involving human participants were reviewed and approved by cohort (1): Ethics was granted by the NHS, Health Research Authority, NRES Committee South East Coast—Brighton and Sussex, Health Research Authority, Skipton House, 80 London Road, London SE1 6LH, UK (REC Reference 15/LO/0218). The second patient cohort was obtained from Discovery Life Sciences (DLS), California, USA. The patient samples are de-identified and publicly available and are thus exempt from the requirement of the Institutional Review Board Ethics Committee (IRB/EC) approval (Exempt Category 4, IRB/EC). All DLS biospecimens are collected under IRB/EC-approved protocols. The samples are procured pursuant to the informed consent provided by the individual, following the general guidelines for informed consent found in the Code of Federal Regulations 45 CFR 46.116. The patients/participants provided their written informed consent to participate in this study.

## Author Contributions

The authors confirm contribution to the paper as follows: study conception and design: CM, MJK, JL, TM, PF, DM, and MR; data collection: CM, JW, HP, DM, and MR; analysis and interpretation of results: CM, JW, MJK, JL, PF, HP, DM, and MR; and draft manuscript preparation: CM, JW, MJK, TM, HP, DM, and MR. All authors contributed to the article and approved the submitted version.

## Conflict of Interest

The authors declare that this study received funding from Randox Laboratories Ltd as part of the Randox Laboratories Ltd – Ulster University PhD Academy Studentship. Randox had the following involvement in the study: analysis of patient samples, statistical analysis, supervision of the project, preparation of the manuscript, and the decision to publish.

JW, MJK, JL, and MR are employees of Randox Laboratories Ltd. but hold no shares in the company. PF is the managing director and owner of Randox Laboratories Ltd. A patent has been filed to protect the biomarker combination disclosed in the manuscript.

The remaining authors declare that the research was conducted in the absence of any commercial or financial relationships that could be construed as a potential conflict of interest.

## Publisher’s Note

All claims expressed in this article are solely those of the authors and do not necessarily represent those of their affiliated organizations, or those of the publisher, the editors and the reviewers. Any product that may be evaluated in this article, or claim that may be made by its manufacturer, is not guaranteed or endorsed by the publisher.
