# Supplementary material for: A Novel Combination of Serum Markers in a Multivariate Model to Help Triage Patients Into “Low-” and “High-Risk” Categories for Prostate Cancer
Source: Front Oncol. 2022 May 19;12:837127. doi: 10.3389/fonc.2022.837127 (PMC9161691; doi:10.3389/fonc.2022.837127)
Supplement: Supplementary file 1 [file DataSheet_1.docx]

**Supplementary 1**

**Inclusion and Exclusion Study Criteria**

| **Inclusion Criteria** | **Exclusion Criteria** |
| --- | --- |
| >18 years  Referral from GP for an abnormal PSA | Active UTI  Men with a PSA < 4 and > 20 ng/ml  Men diagnosed with PCa  Men with a prior or concurrent malignancy  Men who cannot give informed consent |

**Supplementary 2**

| **Marker** | **Role of marker in Prostate Cancer/BPH/LUTS** | **Reference** |
| --- | --- | --- |
| IL-8 | A prospective clinical study of the implications of IL-8 in the diagnosis, aggressiveness and prognosis of prostate cancer | **1** |
| IL-10 | Interleukin 10 (IL-10) Inhibition of Primary Human Prostate Cell-induced Angiogenesis | **2** |
| MCP-1 | Activation of MCP-1/CCR2 axis promotes prostate cancer growth in bone | **3** |
| VEGF | VEGF is associated with the poor survival of patients with prostate cancer: a meta-analysis | **4** |
| IL-1β | IL-1beta and TNF-alpha in prostatic secretions are indicators in the evaluation of men with chronic prostatitis | **5** |
| NSE | Pre-treatment serum level of neuron specific enolase (NSE) as a prognostic factor in metastatic prostate cancer patients treated with endocrine therapy | **6** |
| EGF | The interplay between AR, EGF receptor and MMP-9 signalling pathways in invasive prostate cancer | **7** |
| IL-6 | Tadalafil Treatment Improves Inflammation, Cognitive Function, And Mismatch Negativity of Patients with Low Urinary Tract Symptoms and Erectile Dysfunction | **8** |
| sTNFR1 | New serum biomarkers for prostate cancer diagnosis | **9** |
| CRP | C-reactive protein is significantly associated with prostate-specific antigen and metastatic disease in prostate cancer | **10** |
| D-dimer | Fibrinogen and D-dimer levels in prostate cancer: Preliminary results. | **11** |
| IL-1α | Expression of several cytokines in prostate cancer: Correlation with clinical variables of patients. Relationship with biochemical progression of the malignance | **12** |
| TNFα | IL-1beta and TNF-alpha in prostatic secretions are indicators in the evaluation of men with chronic prostatitis | **5** |
| IL-2 | Prostatic Inflammation in Prostate Cancer: Protective Effect or Risk Factor? | **13** |
| IFNγ | Roles of IFN-γ in tumor progression and regression: a review | **14** |
| IL-4 | Interleukin-4 in patients with prostate cancer | **15** |
| CEA | Elevated carcinoembryonic antigen in patients with androgen-independent prostate cancer | **16** |
| tPSA | The significance of TPSA, free to total PSA ratio and PSA density in prostate carcinoma diagnostics | **17** |
| fPSA | The significance of TPSA, free to total PSA ratio and PSA density in prostate carcinoma diagnostics | **17** |

**References**

1 Roumeguère T, Legrand F, Rassy EE, Kaitouni MI, Albisinni S, Rousseau A, Vanhaeverbeek M, Rorive S, Decaestecker C, Debeir O, Boudjeltia KZ, Aoun F. A prospective clinical study of the implications of IL-8 in the diagnosis, aggressiveness and prognosis of prostate cancer. *Future Sci OA*. 2017 Nov 15;4(2): FSO266. doi: 10.4155/fsoa-2017-0084. PMID: 29379640; PMCID: PMC5778381

2 Stearns ME, Rhim J, Wang M. Interleukin 10 (IL-10) inhibition of primary human prostate cell-induced angiogenesis: IL-10 stimulation of tissue inhibitor of metalloproteinase-1 and inhibition of matrix metalloproteinase (MMP)-2/MMP-9 secretion. *Clin Cancer Res*. 1999 Jan;5(1):189-96. PMID: 9918218

3 Lu Y, Chen Q, Corey E, Xie W, Fan J, Mizokami A, Zhang J. Activation of MCP-1/CCR2 axis promotes prostate cancer growth in bone. *Clin Exp Metastasis*. 2009;26(2):161-9. doi: 10.1007/s10585-008-9226-7. Epub 2008 Nov 11. PMID: 19002595.

4 Zhan P, Ji YN, Yu LK. VEGF is associated with the poor survival of patients with prostate cancer: a meta-analysis. *Transl Androl Urol*. 2013;2(2):99-105.doi:10.3978/j.issn.2223-4683.2013.06.03

5 Robert B. Nadler, Alisa E. Koch, Elizabeth A. Calhoun, Phillip L. Campbell, Denise L. Pruden, Charles L. Bennett, Paul R. Yarnold, Anthony J. Schaeffer. IL-1β and TNF-α in prostatic scretions are indicators in the evaluation of men with chronic prostatitis, *The Journal of Urology*, Volume 164, Issue 1, 2000, Pages 214-218, ISSN 0022-5347

6 Naoto Kamiya, Koichiro Akakura, Hiroyoshi Suzuki, Shinzou Isshiki, Akira Komiya, Takeshi Ueda, Haruo Ito, Pretreatment Serum Level of Neuron Specific Enolase (NSE) as a Prognostic Factor in Metastatic Prostate Cancer Patients Treated with Endocrine Therapy, *European Urology*, Volume 44, Issue 3, 2003, Pages 309-314, ISSN 0302-2838

7 Mandel A, Larsson P, Sarwar M, Semenas J, Syed Khaja AS, Persson JL. The interplay between AR, EGF receptor and MMP-9 signalling pathways in invasive prostate cancer. *Mol Med*. 2018 Jun 27;24(1):34. doi: 10.1186/s10020-018-0035-4. PMID: 30134822; PMCID: PMC6020326.

8 Amparo Urios, Felipe Ordoño, Raquel García-García, Alba Mangas-Losada, Paola Leone, Juan José Gallego, Andrea Cabrera-Pastor, Javier Megías, Juan Fermin Ordoño, Vicente Felipo & Carmina Montoliu. Tadalafil Treatment Improves Inflammation, Cognitive Function, And Mismatch Negativity of Patients with Low Urinary Tract Symptoms and Erectile Dysfunction. *Sci Reports* 9(1); DOI: 10.1038/s41598-019-53136-y

9 Chadha KC, Miller A, Nair BB, Schwartz SA, Trump DL, Underwood W. New serum biomarkers for prostate cancer diagnosis. Clin *Cancer Investig. J*. 2014;3(1):72-79. doi: 10.4103/2278-0513.125802. PMID: 25593898; PMCID: PMC4292911.

10 Lehrer S, Diamond EJ, Mamkine B, Droller MJ, Stone NN, Stock RG. C-reactive protein is significantly associated with prostate-specific antigen and metastatic disease in prostate cancer. *BJU Int*. 2005 May;95(7):961-2. doi: 10.1111/j.1464-410X.2005.05447.x. PMID: 15839913.

11 Selahattin Çalışkan, Mustafa Sungur, Fibrinogen and D-dimer levels in prostate cancer: Preliminary results, *Prostate International*, Volume 5, Issue 3, 2017, Pages 110-112, ISSN 2287-8882,

12 Torrealba N, Rodríguez-Berriguete G, Fraile B, Olmedilla G, Martínez-Onsurbe P, Guil-Cid M, Paniagua R, Royuela M. Expression of several cytokines in prostate cancer: Correlation with clinical variables of patients. Relationship with biochemical progression of the malignance. *Cytokine*. 2017 Jan; 89:105-115. doi: 10.1016/j.cyto.2016.08.008. Epub 2016 Aug 12. PMID: 27527810.

13 Tafuri, A.; Ditonno, F.; Panunzio, A.; Gozzo, A.; Porcaro, A.B.; Verratti, V.; Cerruto, M.A.; Antonelli, A. Prostatic Inflammation in Prostate Cancer: Protective Effect or Risk Factor? *Uro* 2021, 1, 54–59. https://doi.org/10.3390/uro1030008

14 Jorgovanovic, D., Song, M., Wang, L. *et al.* Roles of IFN-γ in tumor progression and regression: a review. *Biomark Res* **8,**49 (2020). https://doi.org/10.1186/s40364-020-00228-x

15 Takeshi U, Sadar MD, Suzuki H, Akakura K, Sakamoto S, Shimbo M, Suyama T, Imamoto T, Komiya A, Yukio N, Ichikawa T. Interleukin-4 in patients with prostate cancer. *Anticancer Res*. 2005 Nov-Dec;25(6C):4595-8. PMID: 16334148.

16 Feuer JA, Lush RM, Venzon D, Duray P, Tompkins A, Sartor O, Figg WD. Elevated carcinoembryonic antigen in patients with androgen-independent prostate cancer. *J Investig. Med*. 1998 Feb;46(2):66-72. PMID: 9549229.

17 Milkovic B, Hadzi-Djokic J, Dzamic Z, Pejcic T. The significance of TPSA, free to total PSA ratio and PSA density in prostate carcinoma diagnostics. *Acta Chir Iugosl*. 2007;54(4):105-7. doi: 10.2298/aci0704105m. PMID: 18595240.
